# Hospital mortality in patients with rare diseases during pandemics: lessons learnt from the COVID-19 and SARS pandemics

**DOI:** 10.1186/s13023-021-01994-z

**Published:** 2021-08-12

**Authors:** Claudia Ching Yan Chung, Wilfred Hing Sang Wong, Brian Hon Yin Chung

**Affiliations:** Department of Paediatrics and Adolescent Medicine, LKS Faculty of Medicine, The University of Hong Kong, 1/F New Clinical Building, Queen Mary Hospital, 102 Pokfulam Road, Pok Fu Lam, Hong Kong SAR

**Keywords:** Rare disease, Pandemic, COVID-19, SARS, Hong Kong, Mortality, Hospital mortality, Risk factor

## Abstract

**Background:**

The threat and experience of pandemics occur differently for different groups. The rare disease population is at particular risk of being further marginalised during pandemics. In this study, our objective was to assess the hospital mortality patterns in the rare disease and the general populations during the coronavirus disease of 2019 (COVID-19) and severe acute respiratory syndrome (SARS) pandemics in Hong Kong.

**Methods:**

All admission records during the COVID-19 pandemic (January 23–August 23, 2020) and SARS pandemic (March 11–June 30, 2003) were extracted from the local public healthcare database. Patients with rare diseases were identified using one or more of the 1084 10th version International Classification of Diseases and Related Health Problems (ICD-10) codes cross-referenced with 467 ORPHAcodes. Hospital mortality patterns were compared in patients with and without COVID-19/SARS infection. Admission records during the same period in 2019 and 2002 were retrieved for comparison.

**Results:**

During the COVID-19 pandemic, 407,219 patients were admitted to one or more of the 43 public hospitals in Hong Kong, of which, 13,894 were patients with rare diseases (3.4%). A total of 4381 and 77 patients from the general and rare disease populations were infected with COVID-19. Rare disease patients had an adjusted 3.4 times odds of COVID-19-related hospital mortality compared with that of the general population (95% C.I. 1.24–9.41). COVID-19-related mortality was almost exclusively seen in patients ≥ 60 years. While age-related increase in mortality was also observed for the general population during the SARS pandemic, the pattern observed in the rare disease population was significantly different, with a 12.5 times higher SARS-related mortality observed in rare disease patients ≤ 18 years than those in the general population (12.5% vs 1.0%). Patients admitted during the same pandemic periods without coronavirus infection had a significantly higher hospital mortality compared with those admitted one year before the pandemic (*p* < 0.001).

**Conclusion:**

This population-based study demonstrated the differential impacts of the COVID-19 and SARS pandemics on the rare disease population. In the era of budget and resource scarcity, this study warrants cautious healthcare planning, with consideration of the rare disease population in healthcare prioritisation.

## Introduction

The coronavirus disease of 2019 (COVID-19) pandemic is an unprecedented global health challenge. Since the outbreak of COVID-19 in Wuhan, China in December 2019, over 127.6 million confirmed cases and 2.7 million deaths were recorded across 222 countries and territories. The outbreak was officially declared as a public health pandemic on March 11, 2020 [1]. Enormous efforts have been made with unprecedented cooperation between governments, policy makers, multidisciplinary members, researchers, and citizens globally to “flatten the curve” of the COVID-19 pandemic. Prospective observational studies have consistently identified old age and chronic major comorbidity to be associated with higher mortality [2–6]. Prioritisation recommendations and guidelines based on scientific and ethical principles underlying COVID-19 vaccination implementation have also been developed to guide allocation of resources, mainly focusing on these groups of patients, and healthcare personnel and essential workers [7].

The World Health Organization (WHO) has acknowledged that the threat and experience of the pandemic occurs differently for different groups [8]. Disregard of the existing differences in healthcare needs and vulnerabilities would pose additional risk of infection to the populations and would undermine the broader COVID-19 response. The rare disease population is systematically excluded in healthcare planning. With rare diseases being heterogeneous and individually rare, patients are often seen to be difficult to manage due to their complex presentations and outcomes of multisystemic involvement and pleiotropic manifestations. Patients with rare diseases require more medical services and resources than the general population in times with and without pandemics. The current COVID-19 pandemic has highlighted and exposed the fundamental and long-standing difficulties and healthcare needs of the rare disease population. In the 7.5 million population in Hong Kong, one in 67 is living with one or more rare diseases [9]. It was found that COVID-19 has affected health status in 46%, mental health in 79%, social life in 92%, daily living in 82%, and financial status in 81% of rare disease patients [10]. With majority of the rare diseases being chronically debilitating or life threatening, patients infected with COVID-19 are at particular risk of being further marginalised.

In response to the emergence of the COVID-19 pandemic and its pandemic potential on the rare disease population, this study sought to assess the hospital mortality patterns in the rare disease and the general populations during the COVID-19 pandemic in Hong Kong. Recognising the differential impacts of pandemics, hospital mortality observed in the rare disease and general populations were compared with that of another coronavirus pandemic, which was the 2003 severe acute respiratory syndrome (SARS) pandemic.

## Methods

### Source of data

In Hong Kong, approximately 90% of inpatient admissions take place in the public hospitals under the management of the Hospital Authority (HA), all of which are available in the Clinical Data Analysis and Reporting System (CDARS) in an unlisted and anonymous manner [9, 11].

### Study population

COVID-19 was first reported on January 23, 2020; whereas SARS was first reported on March 11, 2003 and officially ended in late June 2003 in Hong Kong. This population-based cohort study included all patients who had an inpatient admission at one or more of the 43 hospital(s) under the HA during the period of COVID-19 pandemic (January 23 to August 23, 2020), and during the period of SARS pandemic (March 11 to June 30, 2003). Admission records in the same period in the previous years (January 23 to August 23, 2019; and March 11 to June 30, 2002) were also retrieved for comparison.

Patients with rare diseases were identified using one or more of the 1084 International Classification of Diseases and Related Health Problems, 10th version (ICD-10) codes cross-referenced with 467 ORPHAcodes in the CDARS database, and was previously described elsewhere [9, 12]. Patients admitted with COVID-19 or SARS were identified using ICD-10 in the CDARS database. Other extracted variables included patients’ age at admission, gender, length of stay, and episode death.

### Statistical analysis

Demographic information and study characteristics of the study population were summarised using descriptive statistics. This included the number of subjects, number of admissions, male to female ratio, age at admission, length of stay, number of hospital deaths, and age at episode death. Patient’s age at admission was used for age subgroup analysis (≤ 18, 19–59, ≥ 60). Based on the CDARS data, hospital mortality rate was calculated using episode death, and was assumed to be associated with the same admission.

General association of independent variables against hospital mortality was analysed using logic regression to get the crude odds ratio (OR) and 95% confidence interval (C.I.). Further multivariate logistic regression analyses were performed to show the odds of hospital mortality in patients with rare diseases compared with patients in the general population, adjusted for admission age group (≤ 18, 19–59, ≥ 60), and the presence of a pandemic period. The significant level was set at *p* < 0.05 for two tails. All statistical analyses were conducted using SPSS version 26.0 (IBM SPSS Statistics).

## Results

During the period of January 23 to August 23, 2020, a total of 407,219 patients were admitted to one or more of the public hospitals in Hong Kong, of which, 4381 (1.1%) were COVID-19-related admissions. A total of 13,894 patients with rare diseases were admitted during the same period, of which 77 (0.6%) were admitted with COVID-19 infection. All rare diseases were diagnosed before the COVID-19 pandemic, and were not triggered by the COVID-19 infection. The difference in the frequency of COVID-19 infection between the general and rare disease populations is significant at *p* < 0.001. During the period of March 11 to June 30, 2003, a total of 158,930 patients were admitted, of which 1449 (0.9%) were SARS-related admissions. A total of 5249 patients with rare diseases were admitted during the same period, of which, 50 (1.0%) were admitted were SARS infection. There was no statistically difference in the frequency of SARS infection between the general and rare disease populations. The characteristics of the rare disease and general populations under the COVID-19 and SARS pandemics were summarised in Table 1.

**Table 1:** Rare disease and general population characteristics during SARS and COVID-19 pandemics in Hong Kong

	Rare disease population						General population					
	2002^a^	2003 (SARS pandemic)^a^		2019^b^	2020 (COVID-19 pandemic)^b^		2002^a^	2003 (SARS pandemic)^a^		2019^b^	2020 (COVID-19 pandemic)^b^	
		SARS-related admissions	Non-SARS-related admissions		COVID-19-related admissions	Non-COVID-19-related admissions		SARS-related admissions	Non-SARS-related admissions		COVID-19-related admissions	Non-COVID-19-related admissions
Number of unique headcount	7619	50	5249	18,524	77	13,894	232,059	1449	158,930	543,157	4381	407,219
Male gender (%)	4139 (54.3)	24 (48.0)	3023 (57.6)	10,057 (54.3)	38 (49.4)	7774 (56.0)	107,713 (46.4)	625 (43.1)	76,039 (47.8)	252,713 (46.5)	2157 (49.2)	191,983 (47.1)
Age at admission (mean, median, SD)	38.8, 39.3, 27.83	47.0, 48.0, 21.8	38.9, 40.2, 27.1	46.5, 54.0, 27.3	43.5, 42.3, 25.5	48.9, 56.5, 26.3	51.3, 54.0, 25.6	45.4, 41.8, 20.0	51.9, 55.0, 26.0	57.4, 62.0, 24.7	44.2, 43.5, 19.9	59.0, 63.0, 23.1
Number of admissions	14,304	77	10,401	43,826	95	36,926	368,460	2109	254,068	1,098,497	5277	872,829
Length of stay (mean, median, SD)	8.0, 2.0, 62.6	20.5, 15.5, 16.3	8.0, 2.0, 68.6	5.8, 1.0, 17.0	12.3, 10.0, 9.9	7.4, 4.0, 11.2	6.8, 2.0, 49.6	18.1, 16.0, 14.6	7.8, 2.0, 61.1	4.5, 1.0, 12.0	12.0, 10.0, 9.6	4.0, 1.0, 8.1
Number of episode deaths	730	6	581	1401	5	1020	8478	234	9102	22,644	81	21,510
Age of episode death (mean, median, SD)	63.0, 67.4, 19.4	61.6, 75.2, 30.7	64.5, 68.2, 18.4	69.4, 70.5, 16.7	78.2, 76.5, 7.3	69.7, 70.7, 16.1	73.3, 76.0, 15.4	67.4, 71.9, 17.6	74.3, 77.0, 15.0	78.5, 82.0, 14.3	81.6, 82.6, 9.9	79.0, 82.0, 14.0
Overall mortality rate^c^	9.6	12.0	11.1	7.6	6.5	7.3	3.7	16.1	5.7	4.2	1.8	5.3
Mortality rate by gender (M, F)^c^	12.4, 6.2	16.7, 7.7	13.6, 7.6	9.6, 5.1	5.3, 7.7	9.0, 5.2	4.4, 3.0	20.2, 13.2	6.8, 5.1	4.9, 3.5	2.1, 1.6	6.1, 4.6
Mortality rate by age group (≤ 18, 19–59, ≥ 60)^c^	1.6, 8.0, 18.1	12.5, 3.7, 26.7	1.7, 8.0, 22.0	0.6, 4.6, 14.4	0.0, 0.0, 21.7	0.5, 4.2, 13.3	0.2, 1.2, 7.5	1.0, 7.1, 50.2	0.3, 1.8, 11.7	0.1, 1.1, 7.5	0.0, 0.1, 7.4	0.1, 1.3, 9.2

*COVID-19* coronavirus disease of 2019, *SARS* severe acute respiratory syndrome, *SD* standard deviation

^a^Data retrieved from March 11 to June 30

^b^Data retrieved from January 23 to August 23

^c^Mortality rate was estimated using episode death

### COVID-19-related hospital mortality

Overall, 1.8% (81/4381) of patients with COVID-19 infection died during the same hospital admission, of which, 6.2% (5/81) were patients with rare diseases. The overall COVID-19-related hospital mortality rate in the rare disease patients (6.5%) was 3.6 times higher than that of the general population (1.8%).

Hospital mortality was almost exclusively seen in patients 60 years old or above, with the average age of episode death being 81.6 years for the general population, and 78.2 years for the rare disease population. Hospital mortality over 60 years of age was found to be 21.7% and 7.4% in the rare disease and general populations, respectively.

In deceased patients with COVID-19 infection in the general population, 73.7% had one or more comorbidities. In contrast, only one out of five (20%) deceased patients in the rare disease population had comorbidities unrelated to the rare disease condition. This patient had primary biliary cholangitis, also known as Hanot syndrome (ORPHAcode: 186; ICD-10: K74.3), a rare hepatic disease characterised by autoimmune mediated damage of small intrahepatic bile ducts leading to cholestasis, fibrosis, and cirrhosis, and three other comorbidities: diabetes, heart failure, and osteoporosis. The other four rare disease patients did not have other comorbidities. All of the five rare disease patients who passed away during the same hospital admission had only one rare disease diagnosis pertaining to one rare disease category. Two patients had combined hyperlipidaemia (rare inborn errors of metabolism disease; ORPHAcode: 79211; ICD-10: E78.2), the other three patients were patients with primary biliary cholangitis (rare hepatic disease; ORPHAcode: 186; ICD-10: K74.3), hepatocellular carcinoma (rare neoplastic disease; ORPHAcode: 88673; ICD-10: C22.0), and complex regional pain syndrome type 2 (rare neurologic disease; ORPHAcode: 99994; ICD-10: G56.4) (Table 2).

**Table 2:** COVID-19- and SARS-related hospital mortality in patients with different rare diseases

	Admitted with COVID-19 infection				Admitted with SARS infection			
	Number of admission (%)	Number of people (%)	Number of episode deaths (%)	Mortality rate	Number of admission (%)	Number of people (%)	Number of episode deaths (%)	Mortality rate
Rare abdominal surgical disease	0 (0.0)	0 (0.0)	0 (0.0)	0.0	0 (0.0)	0 (0.0)	0 (0.0)	0.0
Rare bone disease	1 (1.1)	1 (1.3)	0 (0.0)	0.0	0 (0.0)	0 (0.0)	0 (0.0)	0.0
Rare cardiac disease	2 (2.1)	2 (2.6)	0 (0.0)	0.0	3 (3.6)	3 (5.5)	2 (28.6)	66.7
Rare circulatory system disease	0 (0.0)	0 (0.0)	0 (0.0)	0.0	0 (0.0)	0 (0.0)	0 (0.0)	0.0
Rare developmental defect during embryogenesis disease	23 (24.2)	19 (24.7)	0 (0.0)	0.0	4 (4.8)	3 (5.5)	0 (0.0)	0.0
Rare endocrine disease	10 (10.5)	9 (11.7)	0 (0.0)	0.0	3 (3.6)	3 (5.5)	0 (0.0)	0.0
Rare eye disease	2 (2.1)	2 (2.6)	0 (0.0)	0.0	0 (0.0)	0 (0.0)	0 (0.0)	0.0
Rare gastroenterologic disease	0 (0.0)	0 (0.0)	0 (0.0)	0.0	2 (2.4)	2 (3.6)	0 (0.0)	0.0
Rare gynaecologic or obstetric disease	0 (0.0)	0 (0.0)	0 (0.0)	0.0	0 (0.0)	0 (0.0)	0 (0.0)	0.0
Rare haematology disease	19 (20.0)	15 (19.5)	0 (0.0)	0.0	34 (41.0)	24 (43.6)	2 (28.6)	8.3
Rare hepatic disease	1 (1.1)	1 (1.3)	1 (20.0)	100.0	0 (0.0)	0 (0.0)	0 (0.0)	0.0
Rare inborn errors of metabolism	3 (3.2)	3 (3.9)	2 (40.0)	66.7	3 (3.6)	2 (3.6)	0 (0.0)	0.0
Rare immune disease	0 (0.0)	0 (0.0)	0 (0.0)	0.0	0 (0.0)	0 (0.0)	0 (0.0)	0.0
Rare neoplastic disease	7 (7.4)	5 (6.5)	1 (20.0)	20.0	6 (7.2)	5 (9.1)	2 (28.6)	40.0
Rare neurologic disease	22 (23.2)	16 (20.8)	1 (20.0)	6.3	21 (25.3)	9 (16.4)	1 (14.3)	11.1
Rare odontologic disease	0 (0.0)	0 (0.0)	0 (0.0)	0.0	0 (0.0)	0 (0.0)	0 (0.0)	0.0
Rare otorhinolaryngologic disease	9 (9.5)	7 (9.1)	0 (0.0)	0.0	3 (3.6)	2 (3.6)	0 (0.0)	0.0
Rare renal disease	1 (1.1)	1 (1.3)	0 (0.0)	0.0	0 (0.0)	0 (0.0)	0 (0.0)	0.0
Rare respiratory disease	3 (3.2)	3 (3.9)	0 (0.0)	0.0	0 (0.0)	0 (0.0)	0 (0.0)	0.0
Rare skin disease	2 (2.1)	2 (2.6)	0 (0.0)	0.0	0 (0.0)	0 (0.0)	0 (0.0)	0.0
Rare systemic or rheumatologic disease	2 (2.1)	2 (2.6)	0 (0.0)	0.0	4 (4.8)	2 (3.6)	0 (0.0)	0.0
Rare urogenital disease	0 (0.0)	0 (0.0)	0 (0.0)	0.0	0 (0.0)	0 (0.0)	0 (0.0)	0.0
Unique total	95	77^a^	5	6.5	77	50^a^	6^a^	12.0

^a^Some patients had rare disease diagnosis from two or more rare disease categories

Univariate analysis revealed that rare disease patients had a 3.7 times odds of COVID-19-related hospital mortality compared with that of the general population (95% C.I. 1.45–9.37; *p* = 0.006). After adjusting for admission age, it was found that patients with rare diseases had a 3.4 times odds of COVID-19-related hospital mortality compared with those in the general population (95% C.I. 1.24–9.41; *p* = 0.017).

### SARS-related hospital mortality

Overall, 16.1% (234/ 1449) of patients with SARS infection died during same hospital admission, of which, 2.6% (6/234) were patients with rare diseases. The overall SARS-related hospital mortality rate in the rare disease population and general population were 12.0% and 16.1%, respectively. Hospital mortality was found to be the highest in the ≥ 60 years subgroup in both the rare disease and general populations, with the hospital mortality being 26.7% and 50.2%, respectively.

Six rare disease patients who were infected with SARS passed away during the same hospital admission. Five patients (83%) had only one rare disease diagnosis pertaining to one rare disease category. They were patients with alpha-thalassemia (rare haematology disease; ORPHAcode: 846; ICD-10: D56.0), immunodeficiency predominantly affecting antibody production (rare haematology disease; ORPHAcode: 101977; ICD-10: D80.1), familial isolated dilated cardiomyopathy (rare cardiac disease; ORPHAcode: 154; ICD-10: I42.0), acute myeloid leukaemia (rare neoplastic disease; ORPHAcode: 519; ICD-10:C92.0), and trigeminal neuralgia (rare neurologic disease; ORPHAcode: 221091; ICD-10: G50.0). One patient had two rare disease diagnoses: familial isolated dilated cardiomyopathy (rare cardiac disease; ORPHAcode: 154; ICD-10: I42.0) and esophageal carcinoma (rare neoplastic disease; ORPHAcode: 70482; ICD-10: C15.9) (Table 2).

No significant difference in SARS-related hospital mortality was observed between the rare disease and general populations (OR: 0.71; 95% C.I. 0.30–1.68; *p* = 0.434).

### Comparison between COVID-19-related and SARS-related hospital mortality patterns

**Fig. 1:**
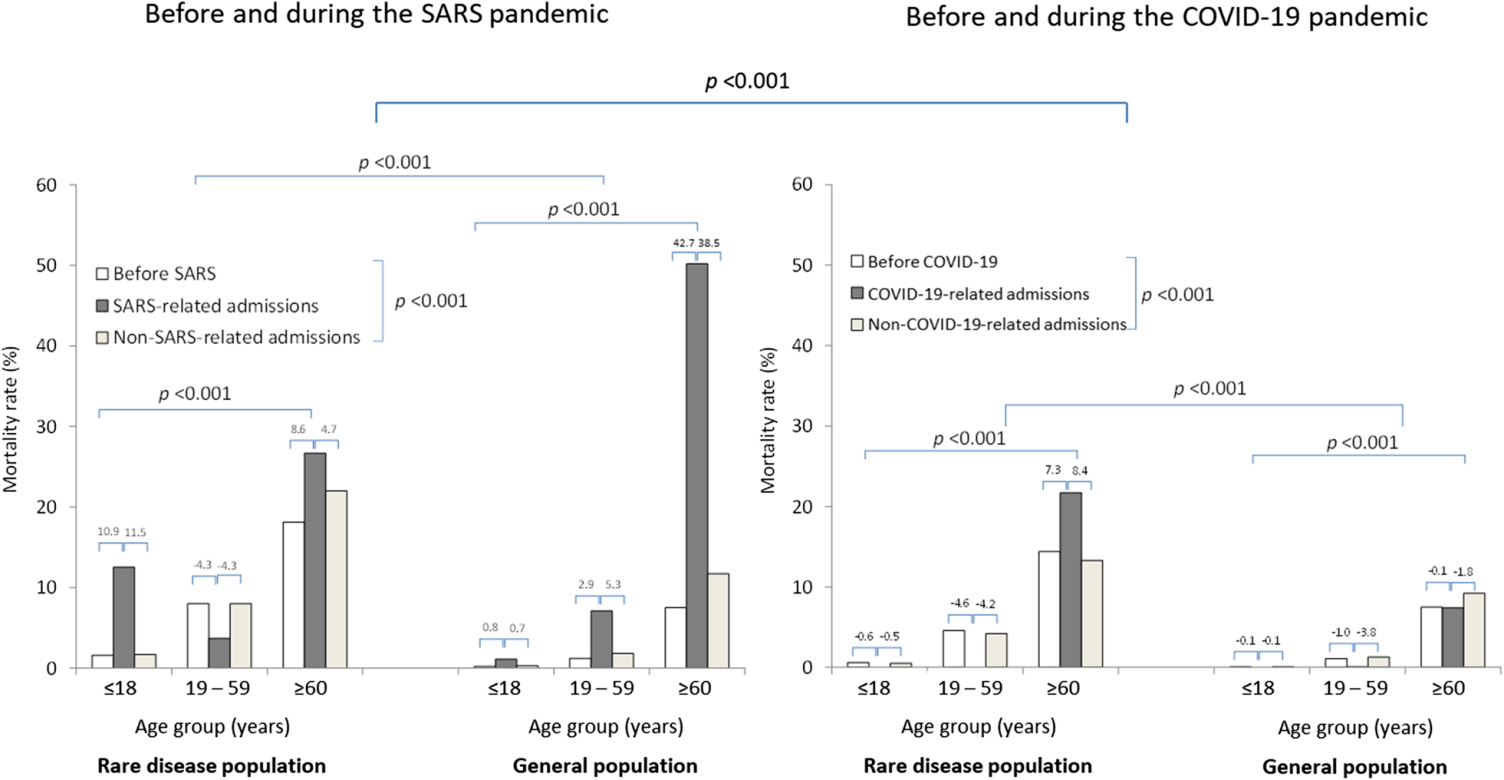
Mortality rate in rare disease and general population before and during SARS and COVID-19 pandemics by age groups

When comparing the hospital mortality patterns during the COVID-19 and the SARS pandemics, significant differences were observed (1) between the COVID-19 and SARS pandemics (*p* < 0.001); (2) between the rare disease population and the general population (*p* < 0.001), and (3) among different age subgroups (≤ 18, 19–59, ≥ 60) (*p* < 0.001) (Fig. 1).

In the general population, the age-related increase in hospital mortality was observed during both the COVID-19 (0.0%, 0.1%, 7.4%) and SARS (1.0%, 7.1%, 50.2%) pandemics using the three age subgroups. The average age of COVID-19-related and SARS-related episode deaths were 81.6 years and 67.4 years, respectively. In contrast, significant differences in hospital mortality patterns were observed in the rare disease population. While older age was also associated with a higher hospital mortality in rare disease patients infected with COVID-19 (0.0%, 0.0%, 21.7%), hospital mortality in rare disease patients infected with SARS did not follow the same pattern (12.5%, 3.7%, 26.7%). SARS-related mortality rate in rare disease patients 18 years old or below (12.5%) was significantly higher than that observed in the general population (1.0%). This was in sharp contrast to the COVID-19 pandemic where mortality was exclusively occurring in patients ≥ 60 years but not in the other age groups.

### Hospital mortality before and during pandemics

In the general population, patients admitted during the COVID-19 and SARS pandemics without coronavirus infection had a higher hospital mortality compared with those admitted in the same period one year before the COVID-19 and SARS pandemics (Table 1). In the rare disease population, similar hospital mortality rate were observed in patients without COVID-19 infection during the pandemic period in 2020, and during the same period in 2019 (7.3% vs 7.6%). Rare disease patients admitted during the SARS pandemic without SARS infection had a higher hospital mortality compared with those admitted in the same period in 2002 (11.1% vs 9.6%).

Patients admitted with COVID-19 infection during the pandemic period (January 23–August 23, 2020) had a 0.7 times odds of hospital mortality compared with those admitted in the same period in 2019 (95% C.I. 0.58–0.89; *p* = 0.002) (Table 3). Patients admitted without COVID-19 infection during the pandemic period had a 1.2 times odds of hospital mortality compared with those admitted in the same period in 2019 (95% C.I. 1.20–1.25; *p* < 0.001), regardless of age and the presence of rare disease.

**Table 3:** Logistic regression model on the association between different variables and the odds of hospital mortality before and during the COVID-19 pandemic

Factors	Crude OR	95% C.I.	*p* value	Adjusted OR^a^	Adjusted 95% C.I.^a^	Adjusted *p* value^a^
Rare disease						
No (general population)	1.00	–	–	1.00	–	–
Yes	1.67	1.60–1.74	< 0.001	2.06	1.98–2.16	< 0.001
Age						
≤ 18	1.00	–	–	1.00	–	–
19–59	8.41	7.52–9.76	< 0.001	8.61	7.42–9.99	< 0.001
≥ 60	60.66	52.43–70.18	< 0.001	62.24	53.79–72.01	< 0.001
Time period						
Admitted one year before pandemic (2019)	1.00	–	–	1.00	–	–
Admitted with COVID-19 infection during pandemic period (2020)	0.44	0.36–0.55	< 0.001	0.72	0.58–0.89	0.002
Admitted without COVID-19 infection during pandemic period (2020)	1.26	1.24–1.29	< 0.001	1.22	1.20–1.25	< 0.001

*C.I.* confidence interval, *COVID-19* coronavirus disease of 2019, *OR* odds ratio

^a^Adjusted for each and all of the variables in the table

In contrast, patients admitted with SARS infection during the pandemic period (March 11–June 30, 2003) had a 8.1 times odds of hospital mortality compared with those admitted in the same period in 2002 (95% C.I. 6.96–9.42; *p* < 0.001), regardless of age and the presence of rare disease (Table 4). Patients admitted without SARS infection in the pandemic period was found to have a 1.6 times odds of hospital mortality compared with those admitted in 2002 (95% C.I. 1.55–1.64; *p* < 0.001).

**Table 4:** Logistic regression model on the association between different variables and the odds of hospital mortality before and during the SARS pandemic

Factors	Crude OR	95% C.I.	*p* value	Adjusted OR^a^	Adjusted 95% C.I.^a^	Adjusted *p* value^a^
Rare disease						
No (general population)	1.00	–	–	1.00	–	–
Yes	2.32	2.18–2.46	< 0.001	2.91	2.73–3.09	< 0.001
Age						
≤ 18	1.00	–	–	1.00	–	–
19–59	4.99	4.34–5.74	< 0.001	5.20	4.52–5.98	< 0.001
≥ 60	31.29	27.30–35.87	< 0.001	33.51	29.22–38.41	< 0.001
Time period						
Admitted one year before pandemic (2002)	1.00	–	–	1.00	–	–
Admitted with SARS infection during pandemic period (2003)	4.78	4.16–5.50	< 0.001	8.10	6.96–9.42	< 0.001
Admitted without SARS infection during pandemic period (2003)	1.57	1.53–1.62	< 0.001	1.60	1.55–1.64	< 0.001

*C.I.* confidence interval, *OR* odds ratio, *SARS* severe acute respiratory syndrome

^a^Adjusted for each and all of the variables in the table

### Association between independent variables and hospital mortality rate

Multivariate logistic regression analysis revealed that rare disease, increasing age, and the presence of a pandemic period were all independently associated with a higher hospital mortality (*p* < 0.001). After adjusting for each and all of the three variables, there was strong evidence that there was a 2.3 times (95% C.I. 2.21–2.37; *p* < 0.001) odds of hospital mortality among patients with rare disease compared to those in the general population, regardless of age and the presence of a pandemic period (Table 5). Patients between 19 and 59 years old, and those 60 years old or above, had a 6.7 times (95% C.I. 6.08–7.45; *p* < 0.001) and 45.9 times (95% C.I. 41.51–50.66; *p* < 0.001) odds of hospital mortality compared with patients 18 years old or below. The presence of a pandemic period was also associated with a higher hospital mortality (adjusted OR: 1.32; 95% C.I. 1.30–1.34; *p* < 0.001).

**Table 5:** Logistic regression model on the association between different variables and the odds of hospital mortality

Factors	Crude OR	95% C.I.	*p* value	Adjusted OR^a^	Adjusted 95% C.I.^a^	Adjusted *p* value^a^
Rare disease						
No (general population)	1.00	–	–	1.00	–	–
Yes	1.85	1.79–1.91	< 0.001	2.29	2.21–2.37	< 0.001
Age						
≤ 18	1.00	–	–	1.00	–	–
19–59	6.54	5.90–7.24	< 0.001	6.73	6.08–7.45	< 0.001
≥ 60	44.35	40.15–48.98	< 0.001	45.86	41.51–50.66	< 0.001
Time period						
One year before pandemic	1.00	–	–	1.00	–	–
During pandemic	1.35	1.33–1.37	< 0.001	1.32	1.30–1.34	< 0.001

*C.I.* confidence interval, *OR* odds ratio

^a^Adjusted for each and all of the variables in the table

## Discussion

This population-based study demonstrated the differential impacts of the COVID-19 and SARS pandemics on different populations. While increasing age remains to be a strong predictor of hospital mortality, the mortality patterns observed in the rare disease population and the general population were significantly different. Rare disease was shown to be one of the risk factors associated with increased hospital mortality. In the current COVID-19 crisis, it points to older patients with rare diseases to be the most vulnerable group. In contrast, while the age-related increase in mortality was also observed for the general population during the 2003 SARS pandemic, SARS-related mortality rate in rare disease patients 18 years old or below were significantly higher than that of the general population. Our study identified sectors of the population that are at a higher risk of mortality, even within the more vulnerable group of patients with rare diseases during pandemics.

Patients with rare diseases had a similar rate of COVID-19 and SARS infection as the general population, but were associated with a significantly higher hospital mortality. Rare diseases are heterogeneous and they often present with complex presentations and outcomes of multisystemic involvement and pleiotropic manifestations. In this study, COVID-19 or SARS infection and its related hospital mortality were not confined to one specific rare disease category. Rare disease as a group, is an independent risk factor for hospital mortality and should be considered in healthcare planning. In particular, for those who were infected with COVID-19, rare disease patients had a 3.4 times odds of hospital mortality compared with those in the general population (95% C.I. 1.24–9.41, *p* = 0.017). This is comparable to other pre-existing comorbidities that were consistently shown to be associated with increased COVID-19-related mortality. For instance, Rastad et al. [13] reported that COVID-19 patients with diabetes mellitus and cardiovascular disease had a 1.6 times odds (95% C.I. 1.14–2.30) and 1.2 times odds (95% C.I. 0.83–1.64) of hospital mortality compared with those without pre-existing comorbidities in a cohort of 2957 patients with COVID-19 in Iran. Gupta et al. showed that coronary artery disease (OR: 1.47; 95% C.I. 1.07–2.02), active cancer (OR: 2.15; 95% C.I., 1.35–3.43), liver dysfunction (OR: 2.61; 95% C.I., 1.30–5.25), and kidney dysfunction (OR: 2.43; 95% C.I. 1.46–4.05) were independently associated with hospital mortality among 2215 adults with COVID-19 who were admitted to intensive care units in the United States [14]. Furthermore, in a cohort of 834 COVID-19 patients ≥ 60 years in Barcelona, Spain, Posso et al. [15] reported an OR of 2.8 (95% C.I. 1.96–3.95) and 1.6 (95% C.I. 1.01–2.55) for patients with chronic kidney disease and heart failure compared with those without, respectively. A meta-analysis by Wang et al. revealed that compared to COVID-19 patients without pre-existing chronic cardiovascular condition, COVID-19 patients who present with either cardiovascular disease or hypertension were associated with an OR of 3.84 (95% C.I. 2.90–5.07) and 2.92 (95% C.I. 2.35–3.64), respectively [16]. The current study provided further evidence to demonstrate that rare disease is also a pre-existing comorbidity that is associated with COVID-19-related hospital mortality. Although no significant differences in hospital mortality were observed between the rare disease and general populations in patients infected with SARS, this could be potentially explained by the much higher case fatality rate associated with SARS-CoV [17].

Patients admitted with COVID-19 infection during the pandemic period had a 0.7 times odds of hospital mortality compared with those admitted in the same period in 2019 (95% C.I. 0.58–0.89; *p* = 0.002). There are four possible reasons behind this. First, patients infected with COVID-19 were on average 13.2 years younger at admission compared with those admitted during the same period in 2019. Older age was an independent risk factor for hospital mortality in times with and without pandemics. Severe COVID-19 outcome and related mortality were strongly associated with advanced age. A younger group of patients suggested that they might be able to recover quicker, and that they are relatively less at risk of COVID-19-related hospital mortality compared with that in older patients. Second, the COVID-19 pandemic has not ended yet. There are patients in both the general and rare disease populations who were still hospitalised during data collection and data analysis. The number of patients being infected with COVID-19 and the number of episode deaths would continue to increase. A COVID-19-related hospital mortality of 1.8% and 6.5% in the general and rare disease populations reflected the impact of the COVID-19 pandemic on the specific study period. Third, with strict public health measures implemented such as the suspension of schools and civil services on January 25 and January 29, 2020, respectively, patients who were infected with COVID-19 were likely to be the more active and healthy group in the community, and admission might not be necessary if they were not infected with COVID-19. Fourth, upon lessons learnt from SARS, the Hong Kong Government has efficiently diverted a lot of resources and manpower to focus on combating COVID-19. Patients who were infected with COVID-19 and were admitted into the hospital would receive high quality care and management, reducing the risk of hospital mortality.

Patients without COVID-19 or SARS infection but were admitted in the pandemic period had a 1.2 and 1.6 times odds of hospital mortality compared with those admitted one year before the pandemic, respectively. Reduction in healthcare service provision under public healthcare measures, and patients’ fear of infection in public areas have caused reduction in health service utilisation during pandemic [10]. During the COVID-19 public health emergency, the Hong Kong Government has efficiently raised the “Preparedness and response plan for novel infectious disease of public health significance” to emergency response level in healthcare settings on January 25, 2020, two days after the first COVID-19 case was confirmed to have spread to Hong Kong [18]. Non-emergency services and elective surgeries were reduced and postponed, and all non-essential visits were suspended. In particular, compared with the inpatient admission number one year before the pandemic, this study showed that the overall inpatient admission has fallen by 26% during the COVID-19 pandemic and by 45% during the SARS pandemic. In a study that assessed the impact of COVID-19 pandemic on rare disease patients without COVID-19 infection in Hong Kong, it was found that health status was impacted in 46% of patients due to reduced service utilisation [10]. The catastrophic impact following cancellation and cessation of healthcare services might have contributed to the overall increased hospital mortality observed during the pandemic period.

Efforts have been made to address the needs of rare disease population during the COVID-19 pandemic, including recently published studies in Hong Kong and in the Asia Pacific Region [10, 19], and reports published within rare disease patient organisations in the local and international settings, such as Rare Disease Hong Kong (RDHK) [20], Asia Pacific Alliance of Rare Disease Organisations (APARDO) [21], and European Organisation for Rare Diseases (EURORDIS) [22]. These studies focused on the perceived impact from the patients’, carers’, or organisation representatives’ perspectives using structured questionnaires, and identified key areas of impacts on patients without COVID-19 infection. To date, the current study is the first and only study that uses a population-based cohort to assess the hospital mortality patterns in rare disease patients with and without COVID-19 or SARS infection. This contributes to the better understanding of the impact of pandemics on this vulnerable group of patients, particularly on hospital mortality patterns. It also warrants the consideration of the rare disease population during healthcare planning.

Our study has several strengths. This is a population-based cohort study that included over 90% of all inpatient admissions in Hong Kong during the study period. It assessed the hospital mortality patterns observed in both the rare disease and general populations, and demonstrated the differential impacts during two pandemics: COVID-19 and SARS. There are, however, several limitations acknowledged. First, this study did not account for other comorbidities when evaluating the association between independent risk factors and mortality rate. Second, this study did not evaluate the associated therapies that could possibly be an aggravating factor. Third, some patients admitted during the COVID-19 pandemic were still hospitalised and receiving ongoing care, overall mortality rate and mean length of stay in both the rare disease and general populations during this period might be underestimated. Last, mortality rate in the rare disease population infected with COVID-19 or SARS were based on a small number of episode death. However, rare diseases are individually rare, and the number of rare disease patients with COVID-19 or SARS infection were even rarer, it was not surprised to see an even smaller number of rare disease patients who were infected and passed away during the pandemics. The identification of rare disease patients using the comprehensive CDARS database was considered to be the best option, and was invaluable to provide empirical and rapid evidence especially during pandemics. In fact, the overall number of episode deaths for those infected with COVID-19 or SARS were relatively low in Hong Kong, partly due to the efficient and effective public health measures implemented by the Hong Kong Government. Among all COVID-19-related episode deaths, 6.2% were attributed by patients with rare diseases, demonstrating the significance of the rare disease population.

## Conclusion

This population-based cohort study adds to available literature by demonstrating the differential impacts of the COVID-19 and SARS pandemics on the rare disease and general populations in Hong Kong. It also gives an opportunity to address structural deficits that have been consistently and disproportionately affecting the rare disease population. As highlighted by the WHO, the threat and experience of the pandemic occurs differently for different groups. Real-time data analysis by age group within different populations can be of consideration when developing prioritisation recommendations or guidelines for healthcare planning in the future. Taken together, this study underscores the importance of using near real-time hospital data in times of pandemics and warrants cautious and careful healthcare planning, with consideration of the rare disease population in healthcare prioritisation. Upon lessons learnt from SARS and now the COVID-19 pandemic, our challenge is not to control the pandemic to return to the previous normal, but to improve our preparedness based on scientific evidence and ethical principles to respond to the possible emergence of another new outbreak in the future.

## Data Availability

The data that support the findings of this study are not publicly available. Data are available from the Hong Kong Hospital Authority but restrictions apply to the availability of these data, which were used under license for the current study. Data are however available from the authors upon reasonable request and with permission from the Hong Kong Hospital Authority.
